# Visualization of Network Target Crosstalk Optimizes Drug Synergism in Myocardial Ischemia

**DOI:** 10.1371/journal.pone.0088137

**Published:** 2014-02-05

**Authors:** Xiaojing Wan, Jia Meng, Yingnan Dai, Yina Zhang, Shuang Yan

**Affiliations:** 1 Department of Geriatrics, the Fourth Affiliated Hospital of Harbin Medical University, Harbin, China; 2 Department of Geriatrics, the Second Affiliated Hospital of Harbin Medical University, Harbin, China; 3 Department of Cardiology, the Fourth Affiliated Hospital of Harbin Medical University, Harbin, China; 4 Department of Endocrinology, the Fourth Affiliated Hospital of Harbin Medical University, Harbin, China; Emory University, United States of America

## Abstract

Numerous drugs and compounds have been validated as protecting against myocardial ischemia (MI), a leading cause of heart failure; however, synergistic possibilities among them have not been systematically explored. Thus, there appears to be significant room for optimization in the field of drug combination therapy for MI. Here, we propose an easy approach for the identification and optimization of MI-related synergistic drug combinations via visualization of the crosstalk between networks of drug targets corresponding to different drugs (each drug has a unique network of targets). As an example, in the present study, 28 target crosstalk networks (TCNs) of random pairwise combinations of 8 MI-related drugs (curcumin, capsaicin, celecoxib, raloxifene, silibinin, sulforaphane, tacrolimus, and tamoxifen) were established to illustrate the proposed method. The TCNs revealed a high likelihood of synergy between curcumin and the other drugs, which was confirmed by *in vitro* experiments. Further drug combination optimization showed a synergistic protective effect of curcumin, celecoxib, and sililinin in combination against H_2_O_2_-induced ischemic injury of cardiomyocytes at a relatively low concentration of 500 nM. This result is in agreement with the earlier finding of a denser and modular functional crosstalk between their networks of targets in the regulation of cell apoptosis. Our study offers a simple approach to rapidly search for and optimize potent synergistic drug combinations, which can be used for identifying better MI therapeutic strategies. Some new light was also shed on the characteristic features of drug synergy, suggesting that it is possible to apply this method to other complex human diseases.

## Introduction

Myocardial ischemia (MI) is an extremely common cardiovascular disease in modern society, and is the most common cause of mortality in the world [1]. The usual pathological cause of MI is coronary artery disease, which presents the heart muscle with a dilemma: on the one hand, a reduced blood supply and, on the other hand, the need for flexible blood delivery in the course of daily activities. Several pathological mechanisms may be implicated in the pathogenesis of myocardial dysfunction in MI, including myocardial apoptosis (programmed cell death) [2], massive release of reactive oxygen species (ROS) [3], aberrant cardiac electrophysiology [4], and myocardial fibrosis [5]. Therefore, in clinical practice, multidrug intervention is generally considered a standard approach in the current management of MI [6], [7]. In comparison with monotherapy, the potential advantages of multidrug therapy are: (1) relevant therapeutic selectivity can be significantly improved while drug dose can be proportionally reduced and (2) adverse drug reactions will be avoided as much as possible [8], [9]. For instance, joint use of the hydroxymethyl glutaryl coenzyme A (HMG-CoA) reductase inhibitor simvastatin and the cholesterol absorption inhibitor ezetimibe has been proven to be an effective approach for controlling low-density lipoprotein cholesterol (LDL-C) levels. This approach is capable of avoiding excessive use of statins and related serious adverse reactions such as rhabdomyolysis [10]. Potential synergy between lipid-lowering drugs of different classes also plays an important role [11].

The complex pathology of MI indicates that protective effects can be conferred by using drugs that have different mechanisms of action [12]–[19]. Nonetheless, until now, only a few experimental studies of such synergistic effect on MI could be found in the literature [20]–[22]. This situation implies rarity of drug synergism in the real world [23] and the lack of effective methods for identifying drug synergy or tools that are applicable to MI. Existence of a large number of drugs with validated benefits in MI and the advantages of synergistic drug therapeutics prompted us to systematically explore the general characteristics of possible synergy between MI-related drugs, especially within the framework of a network of drug targets as discussed previously [24]. Each drug has a unique network of targets, numbering in the dozens, of proteins and other molecules. Working within the hypothesis that functional crosstalk (or overlap) between the networks corresponding to 2 drugs implies synergy between these drugs, we proposed an easy-to-use network biology approach that allows a researcher to intuitively identify and optimize synergistic drug combinations for MI treatment in a visual way. In this work, our *in vitro* experiments confirmed that more dense functional crosstalk between networks of drug targets (and modular intervention in cell apoptosis by means of the relevant drugs) can open up greater possibilities for finding synergy in myocardial protection.

Overall, our present study established an easy-to-use method to identify an optimized drug combination for MI treatment by visualizing the functional crosstalk between the networks corresponding to these drugs. This approach also provided novel insight into the prerequisites of drug synergism from the network perspective. Furthermore, avoidance of parameter calculations, simplification of procedures, and limited need for biological information makes practical application of this method feasible. In this study, 8 drugs (4 natural compounds, including curcumin, capsaicin, silibinin, and sulforaphane, the nonsteroidal anti-inflammatory drug celecoxib, the immunosuppressant tacrolimus, and 2 selective estrogen receptor modulators, raloxifene and tamoxifen) were chosen as examples for illustrating the method. We believe, however, that our approach will be effective with other drug combinations as well.

## Results

### Target crosstalk networks (TCNs) highlight functional crosstalk within the network of targets of different drugs

**Figure 1:**
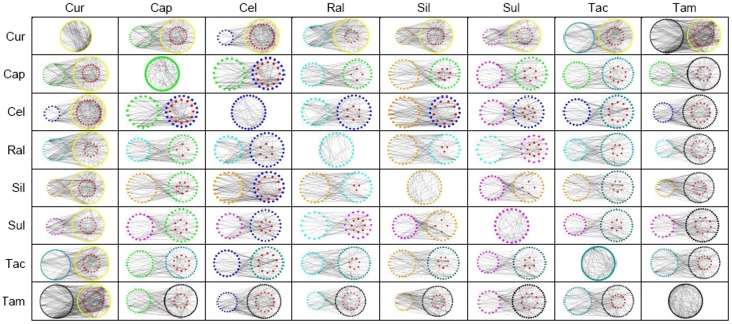
Visualization of target crosstalk networks (TCN) of random drug combinations that might be beneficial in myocardial ischemia (MI). Nodes of differentiated colors represent network targets that are associated with different drugs. The red node means that a network target is commonly associated with 2 combined drugs in each TCN. In the construction of TCN, isolated nodes were deleted in target association networks of single drugs; however, in TCNs, those nodes should also have been removed if the represented proteins had no functional interaction with any network target belonging to another drug. A dense TCN indicates the presence of close functional associations between the respective targets of the combined drugs. Cur: curcumin; Cap: capsaicin; Cel: celecoxib; Ral: raloxifene; Sil: silibinin; Sul: sulforaphane; Tac: tacrolimus; Tam: tamoxifen.

In general, a single drug could simultaneously target or be functionally associated with multiple proteins, which tend to interact with each other or additional proteins and form a network-like functional association of drug targets [25]. Thus, the proteins that are associated with a given drug could be thought of as its network of targets (if the genome-wide scope of the network of interactions of human proteins was considered a background). Based on the concept of a drug’s network of targets, we established target association networks of 8 drugs by retrieving trusted chemical-protein interaction data from the STITCH 3.1 database [25] and applying the Cytoscape [26] plug-in Bisogenet [27]. The 8 drugs that were included in the present study were: 4 natural compounds, curcumin, capsaicin, silibinin, and sulforaphane; the nonsteroidal anti-inflammatory drug celecoxib; the immunosuppressant tacrolimus; and 2 selective estrogen receptor modulators, raloxifene and tamoxifen. Figure 1 shows the constructed target association networks for each of the drugs. Because curcumin and tamoxifen possess approximately 150 targets in their networks, the largest networks were constructed for these two drugs compared to the others. For instance, sulforaphane was assigned a relatively small network because it functionally interacts with only 43 targets.

Despite big differences in network size, no obvious impact of the size could be detected on the subsequently established TCNs of random pairwise drug combinations (Figure 1). Curcumin tended to have denser TCNs with the other drugs, but tamoxifen did not. A denser TCN indicates the presence of close functional associations between the respective targets of the combined drugs and suggests a greater possibility of synergism between the 2 drugs, compared to more sparse TCNs [28]. This result visually indicates potential extensive synergy of curcumin with the other drugs. In addition, we found that the combination of celecoxib and silibinin produced a dense TCN, whereas the drug pair of celecoxib and sulforaphane did not. Silibinin had 47 targets in its network, which was comparable with the number of targets for sulforaphane (43 targets), again showing the limited impact of the number of targets on the interaction density of TCNs.

### Drug combinations with dense TCNs provided synergistic protection against MI

**Figure 2:**
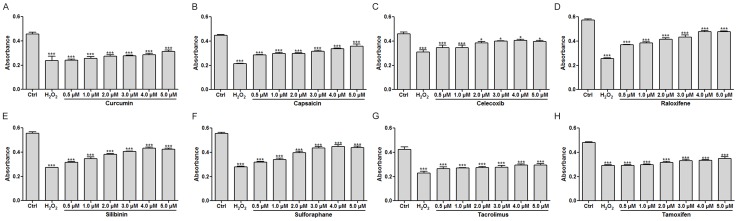
Assessment of a protective effect of stand-alone drugs (in the concentration range from 0.5 to 5.0 µM) against H_2_O_2_-induced myocardial apoptosis in ventricular cardiomyocytes of neonatal rats. A–H. Results of the MTT assay for stand-alone administration of curcumin, capsaicin, celecoxib, raloxifene, silibinin, sulforaphane, tacrolimus, or tamoxifen. *p<0.05, ***p<0.001 compared to the control group; n = 6.

First, the protective effect of each drug was separately investigated in an *in vitro* model of H_2_O_2_-induced myocardial injury in ventricular cardiomyocytes of neonatal rats. A positive association between the dose and effect could be found for all of the 8 drugs in the concentration range from 0.5 to 5.0 µM (Figure 2). Raloxifene at 4.0 µM exhibited the best protective effect against H_2_O_2_-induced myocardial injury among the 8 drugs studied here, although complete recovery was still not achieved in terms of cell viability (Figure 2D). It should be noted that a strong significant benefit should not be expected to be achieved by simply increasing the dose of a drug (Figure 2G and H).

**Figure 3:**
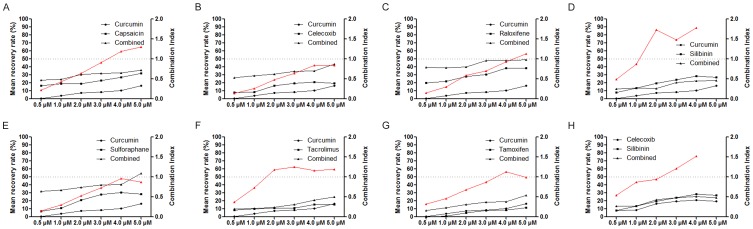
Evaluation of a protective effect of 2-drug combinations (in the concentration range from 0.5 to 5.0 µM) against H_2_O_2_-induced myocardial apoptosis in ventricular cardiomyocytes of neonatal rats. A–G. Combination effects of curcumin with one of the other drugs at a dose ratio of 1∶1; H. The combination effect of celecoxib and silibinin at a dose ratio of 1∶1. The red line with triangles shows the results of calculation of the combination index. A combination index of less than 1.0 indicates potential synergism of the 2 drugs in question.

Based on the TCN results, eight random 2-drug combinations among the 28 combinations were selected for *in vitro* experimental validation, according to dense TCNs that imply synergistic protection against ischemia (Figure 3). After the raw absorbance values in the 3-[4,5-dimethylthiazol-2-yl]-2,5-diphenyl tetrazolium bromide (MTT) assay were transformed into recovery rates (see **Materials and Methods**), we calculated the synergy parameter combination index (CI) for each drug combination at different concentrations. A value of CI less than 1.0 indicates synergy between the drugs in question [28]. At the concentration of 0.5 µM, curcumin did indeed synergistically interact with the other drugs to varying extents (Figure 3A–G). In particular, although raloxifene at 4.0 µM alone could provide considerable recovery in cell viability, addition of curcumin could dramatically reduce the equivalent concentration of the former, implying strong synergism between the 2 drugs according to the CI value (Figure 3C). Similarly, we also experimentally verified the synergistic relationship between celecoxib and silibinin at the low concentration of 0.5 µM (Figure 3H).

**Figure 4:**
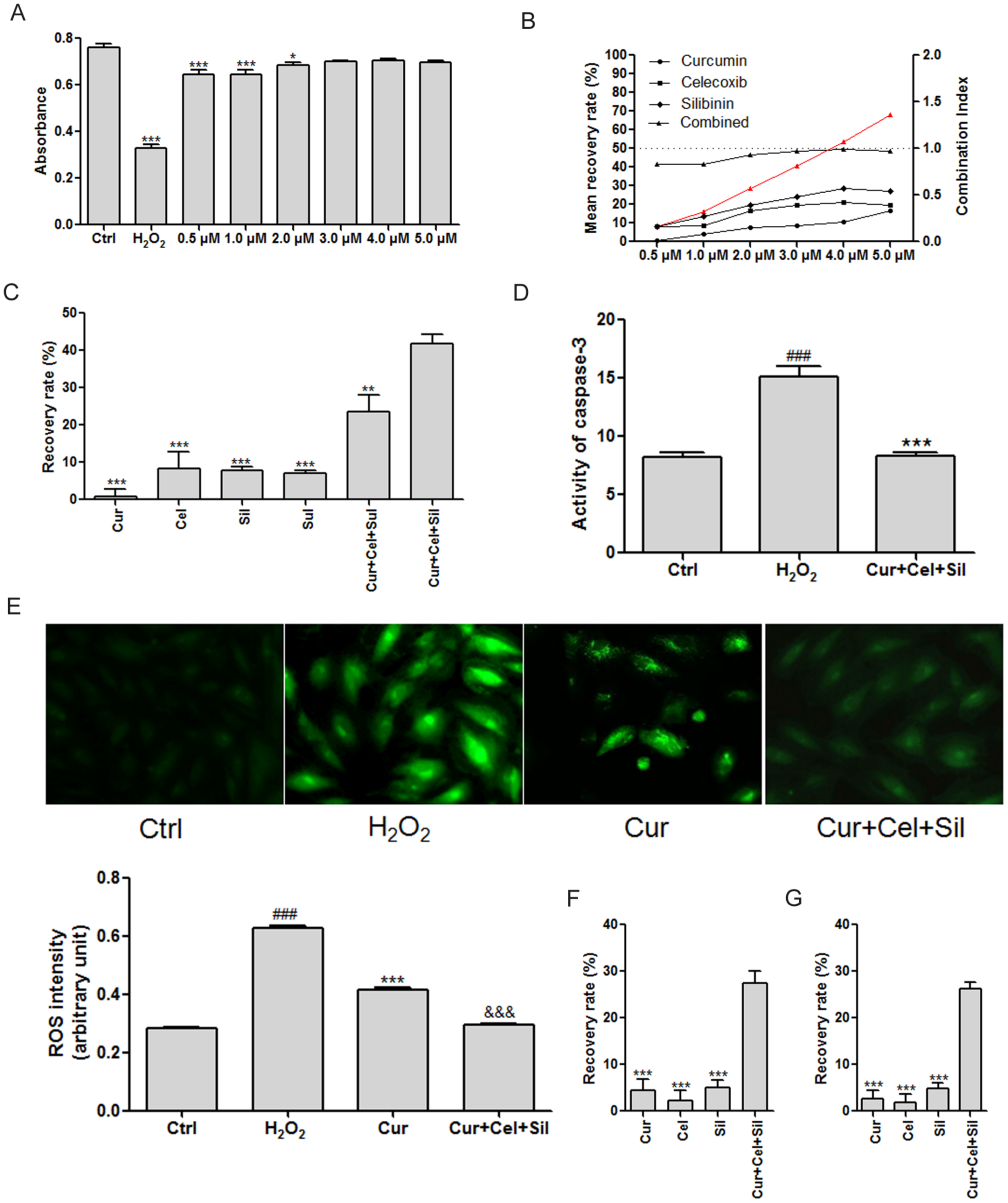
Evaluation of protective effects of 3-drug combinations against H_2_O_2_-induced myocardial injury in ventricular cardiomyocytes of neonatal rats. A. Cell viability recovery provided by the 3-drug combination of curcumin, celecoxib, and silibinin at different concentrations. *p<0.05, ***p<0.001 compared to the control group; n = 6; B. Results of calculation of the combination index of the 3-drug combination of curcumin, celecoxib, and silibinin at different concentrations. The red line with triangles shows the result of calculation of the combination index; C. Comparison of different drugs and drug combinations in the gain of the recovery rate of cell viability in relation to H_2_O_2_-induced myocardial injury. **p<0.01, ***p<0.001 compared to the Cur+Cel+Sil group; n = 6; D. Results of measurement of caspase 3 activity. ^###^ *p*<0.001 compared to the control group; ***p<0.001 compared to the H_2_O_2_ group; n = 3; E. Results of measurement of the level of reactive oxygen species. ^###^ *p*<0.001 compared to the control group; ***p<0.001 compared to the H_2_O_2_ group; ^&&&^ *p*<0.001 compared to the Cur group; n = 6. F. The synergistic effect of drugs in the gain of the recovery rate of cell viability against hypoxia-induced myocardial injury. ***p<0.001 compared to the Cur+Cel+Sil group; n = 6; G. The synergistic effect of drugs on cell viability after H_2_O_2_ treatment. ***p<0.001 compared to the Cur+Cel+Sil group; n = 6; Cur: curcumin; Cel: celecoxib; Sil: silibinin; Sul: sulforaphane. A concentration of 0.5 µM was used with each drug; the dose ratio was 1∶1∶1.

In the present study, we also designed a pattern of 3-drug combinations according to the results of TCNs. Because curcumin, celecoxib, and silibinin have a synergistic relationship, we tested them simultaneously on H_2_O_2_-treated cardiomyocytes. Both strong synergy and a significant protective effect were observed (Figure 4A and B). By contrast, when silibinin was replaced by sulforaphane (which failed to form a dense TCN with celecoxib), we could not observe the same synergistic effect. These data support the predictive utility of TCN in the search for and optimization of synergistic combinations of drugs (Figure 4C). Furthermore, an *in vitro* experiment confirmed significant inhibition of activity of caspase 3 by the optimized 3-drug combination consisting of curcumin, celecoxib, and silibinin (Figure 4D). The synergistic combination could alleviate oxidative stress better than curcumin alone (Figure 4E). In addition, we successfully validated the potent synergistic effect of curcumin, celecoxib, and silibinin under hypoxic conditions and after H_2_O_2_ treatment (Figure 4F and G).

### Functional module analysis confirmed the drug synergism predicted by TCN

**Figure 5:**
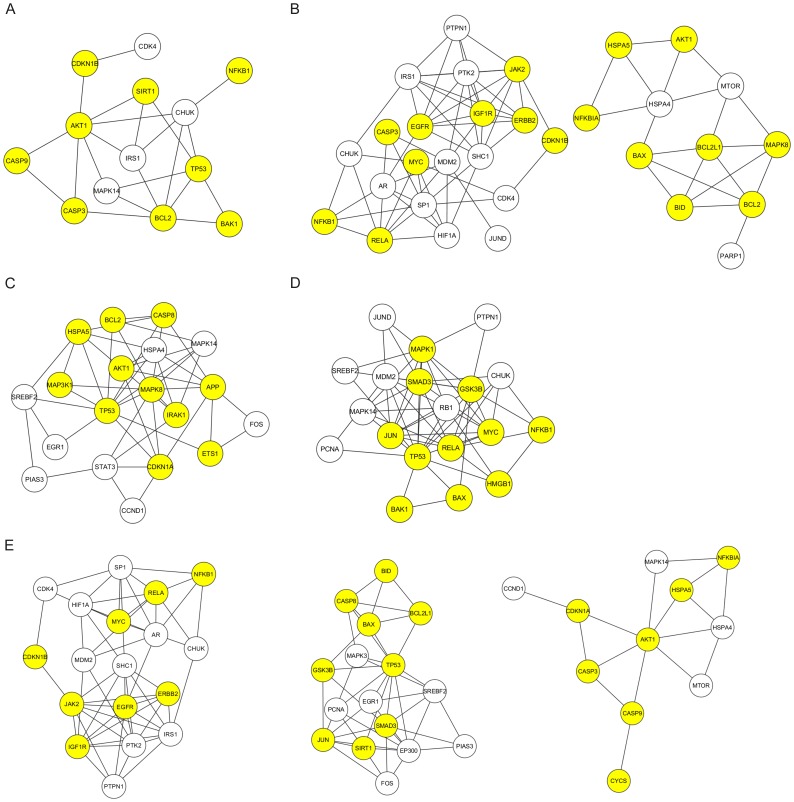
Illustration of biological function modules that were affected by drug combinations and were significantly implicated in the regulation of cell apoptosis (false discovery rate <0.05). A. An apoptosis regulatory module affected by the 2-drug combination of celecoxib and silibinin; B. Apoptosis regulatory modules affected by the 2-drug combination of curcumin and silibinin; C. An apoptosis regulatory module affected by the 2-drug combination of curcumin and celecoxib; D. An apoptosis regulatory module affected by the 3-drug combination of curcumin, celecoxib, and sulforaphane; E. Apoptosis regulatory modules affected by the 3-drug combination of curcumin, celecoxib, and silibinin; the yellow node represents a network target that is involved in the regulation of cell apoptosis.

Dense functional protein association intrinsically leads to the generation of a functional module, in which many proteins are interacting and are collectively responsible for fine adjustment of complex biological processes. Here we tested whether the synergistic effect of a drug combination was the result of action of these close-knit modules, i.e., the result of modular intervention of complex biological processes, such as regulation of cellular apoptosis. Our functional module analysis revealed that curcumin, celecoxib, silibinin, and sulforaphane did not target any functional module that was significantly involved in cell apoptosis regulation (false discovery rate > 0.05) when their respective target association networks were disassembled (see **Materials and Methods**). Nevertheless, when the target association networks were combined for this analysis, the Cytoscape plug-in ClusterMaker [29] and the functional annotation tool of DAVID [30] did identify apoptosis regulatory modules. These data showed better intervention of synergistic drug combinations on the apoptotic state of ischemic cardiomyocytes (Figure 5). It is noteworthy that addition of silibinin to the combination of curcumin and celecoxib generated more apoptosis regulatory modules compared with the alternative replacement with sulforaphane (Figure 5D and E). This observation can explain the significant difference between the two 3-drug combinations in recovering cellular viability from H_2_O_2_-induced ischemic injuries (Figure 4C). Furthermore, our results revealed that compared to a single drug, a synergistic drug combination was more likely to cause an overall change in a functional module (Figure S1). For example, the protein products of *BAX*, *BCL2L1*, *BID*, *CASP8*, and *TP53* are functionally associated, implying close interactions among them in apoptosis regulation. Both the STITCH 3.1 database search results and our experiment confirmed that *BAX*, *BCL2L1*, and *TP53* were common targets of curcumin, that celecoxib simultaneously targeted *BAX*, *CASP8*, and *TP53*, and that silibinin was associated with *BID* and *TP53*. We found that a multiple-drug combination, as opposed to a single drug, caused the most significant alterations in gene expression. This finding was consistent with the potent myocardial protection provided by the synergistic combination composed of curcumin, celecoxib, and silibinin (Figure 4).

## Discussion

MI-related cardiovascular diseases still remain the leading cause of death in the world [1]. This epidemiological finding suggests that treatment of this disease leaves much to be desired. This notion is especially relevant to and practical for MI, in which pharmacotherapy is still dominant in the disease management [6], [7] and increasing numbers of drugs and chemicals have shown positive therapeutic potential [12]–[19]. For the purpose of rational selection and optimization of therapeutic drugs, the synergistic mode of action of drugs should be given priority when multidrug therapy is indicated [6], [7]. This is because the synergistic approach can fully exploit the intrinsic biological system factors such as network robustness, redundancy, and crosstalk [23]. In a biological system, a functional association among genes, proteins, or both, constitutes complexity of the organization of biological activities. Therefore, it is acceptable to subscribe to the point of view that targeting isolated genes or proteins with irrelevant biological functions will not contribute to substantive drug synergy [24]. Conversely, direct action on the interconnected factors that are responsible for network stability in a biological system (instead of taking a detour) may be essential for obtaining a therapeutic effect because of “the whole is greater than the sum of the parts” principle [9]. With full comprehension of the network-driven drug synergy, we proposed a hypothesis that the potential for drug synergy could be visualized as a target crosstalk network, namely TCN, which intuitively demonstrates the functional linkages between individual drugs’ networks of targets.

Our *in vitro* experiments clearly validated the ischemic protection conferred by the MI-related drugs that were involved in the present study; these data are consistent with the results of other studies [12]–[19]. On the negative side, only a limited gain in recovered cell viability was observed with increasing drug concentrations (Figure 2). We can speculate that the inherent robustness of the biological system dampened further effects of drugs [23]. Thus, the belief in a substantial increase of a drug dose in order to achieve desired effect is not merited, because potential adverse drug reactions may be unavoidable [31]. The alternative approach is to attempt to weaken the biological robustness that attenuates the drug action. Drug synergy seems to be a good choice in this regard [32]. Accordingly, simple operations in Cytoscape were performed to establish the TCNs of MI-related drug combinations with the explicit goal of the identification and optimization of drug combinations that were synergy-powered (Figure 1). By comparing the interaction density of TCNs, one can intuitively gauge sources of potential synergy. Curcumin could be considered the ‘life of the party’, able to show synergy with most other drugs, as evidenced by high densities of TCN interactions. Our finding is consistent with the considerable evidence of curcumin-based drug synergies [33]–[42]. In our study, we experimentally verified the synergistic ability of curcumin (Figure 3). Strong synergistic relationships with the other 7 drugs were found at the concentration of 0.5 µM. For example, obvious enhancement of the protective effect of celecoxib could be seen when coadministered with curcumin (Figure 3B). This result is in line with the interaction density of the corresponding TCNs.

Based on the results of TCNs, we further optimized the curcumin-based synergistic drug combination by performing the assays of cell viability, caspase 3 activity, and ROS content (Figure 4). Ultimately, an optimized 3-drug combination was found, which consists of curcumin, celecoxib, and silibinin. Our results suggest that pretreatment with the combination of curcumin, celecoxib, and silibinin effectively reduced oxidative stress in H_2_O_2_-treated cardiomyocytes. By contrast, we did not expect excellent performance from a different 3-drug combination—curcumin, celecoxib, and sulforaphane (Figure 4C)—because of the poor target crosstalk between celecoxib and sulforaphane (Figure 1). A poor target crosstalk indicates a lack of close functional associations between the targets of the combined drugs. Functional module analysis revealed that the failure to influence apoptosis-regulatory modules was the potential mechanism, in contrast to the 3-drug combination of curcumin, celecoxib, and silibinin (Figure 5). These data indicate that an intervention that targets a biological module instead of isolated proteins enhances the ability of drugs to overcome biological robustness [43], [44]. In our study, we confirmed that synergistic drug combinations could influence apoptosis regulatory modules, which is a difficult task for a single drug (Figure S1). This finding demonstrates a distinct advantage of multidrug therapy, especially a synergy-powered multidrug therapy, over monotherapy.

In conclusion, our study provided a concise network-based method that can be easily used in designing and optimizing synergistic combinations of drugs with validated therapeutic potential in MI. Innovative development of synergistic drug combinations may lead to better therapeutic strategies for this disease. Because of the rapid accumulation of experimentally validated data on drug-target interactions, we believe that our method can also be applied to exploring synergistic drug combinations for the treatment of other complex diseases.

## Materials and Methods

### Cell culture and drug treatment

In this study, the use of animals complied with the Guide for the Care and Use of Laboratory Animals published by the US National Institutes of Health (NIH Publication, No.85-23, revised 1996), and was preapproved by the experimental animal ethic committee of the Harbin Medical University, China (Animal Experimental Ethical Inspection Protocol, No. 2009104). Neonatal rat ventricular cardiomyocytes were isolated from 1- to 2-day-old Sprague-Dawley (SD) rats. Briefly, hearts were quickly minced and digested with 0.25% trypsin. The cell suspensions were centrifuged at 2000 rpm for 180 s, then the cells were incubated for 2 h in the medium consisting of Dulbecco’s Modified Eagle Medium (DMEM), 10% fetal bovine serum, 100 U/mL penicillin, and 100 U/mL streptomycin. Cardiomyocytes were collected and then grown in DMEM for another 48 h.

Curcumin, capsaicin, celecoxib, raloxifene, silibinin, sulforaphane, tacrolimus, and tamoxifen were all purchased from Sigma-Aldrich Co., USA. After incubation with the drugs, cardiomyocytes were cultured in a serum-free medium for 12 h prior to being exposed to 100 µM of H_2_O_2_ for another 2 h in a humidified incubator in the presence of 95% O_2_ and 5% CO_2_. The concentrations of all the drugs studied here were in the range from 0.5 to 5.0 µM. Furthermore, 1∶1 or 1∶1∶1 concentration ratios were used to investigate the synergistic effects of 2- or 3-drug combinations against H_2_O_2_-induced myocardial apoptosis on cardiomyocytes in the concentration range from 0.5 to 5.0 µM.

### The cell viability assay

Viability of cells was assessed by measuring mitochondrial dehydrogenase activity, using the colorimetric MTT assay, based on the fact that viable cells rather than dead cells can reduce MTT. Briefly, the cultured cardiomyocytes were plated in 96-well plates. After drug and H_2_O_2_ treatment, the cells were incubated with 10 µL MTT (0.5 mg/mL) at 37°C for 4 h. In addition, the cytoprotective effect of drugs was explored by adding drugs after a 10-min pretreatment with 100 µM of H_2_O_2_. After that, the cardiomyocytes were exposed to 100 µM of H_2_O_2_ for another 2 h in a humidified incubator in the presence of 95% O_2_ and 5% CO_2_. We also investigated the effect of the drugs on recovery of cell viability under hypoxic conditions by exposing the cells to hypoxia (1% O_2_, 94% N_2_, 5% CO_2_) for 24 h in a modular incubator. The purple formazan crystal was dissolved with 100 µL of dimethyl sulfoxide (DMSO) and added to the cells. The absorbance was measured on a spectrophotometer (Tecan Group Ltd.; Switzerland) at 570 nm. To calculate the values of the combination index (CI) for drug combinations as described before [28], we transformed the raw absorbance values into a percentile parameter recovery rate of cell viability, which was calculated as a difference between the absorbance value in a drug administration group and the mean absorbance value in an H_2_O_2_ group divided by the mean absorbance value in the control group.

### The caspase 3 activity assay

Caspase 3 activity of cardiomyocytes was determined using the Caspase 3 Activity Assay Kit (Beyotime Institute of Biotechnology; China), which is based on the ability of caspase 3 to change acetyl-Asp-Glu-Val-Asp p-nitroanilide (Ac-DEVD-pNA) into the yellow formazan product p-nitroaniline (pNA). The detailed analysis procedure is described in the manufacturer’s protocol (Beyotime Institute of Biotechnology; China). Caspase 3 activity was calculated as a ratio of p-nitroanilide content to the total protein amount.

### The reactive oxygen species (ROS) assay

A change in intracellular ROS levels in cardiomyocytes was detected using the ROS assay kit (Beyotime Institute of Biotechnology; China) by measuring the oxidative conversion of cell-permeable 2V,7V-dichlorofluorescein diacetate (DCFH-DA) to fluorescent dichlorofluorescein (DCF) on a fluorospectrophotometer (F4000; Japan). Following the manufacturer’s instructions, the results were determined at an excitation wavelength of 488 nm and at an emission wavelength of 535 nm. The ROS intensity was expressed in arbitrary units.

### Quantitative reverse transcription-polymerase chain reaction (qRT-PCR)

RNA samples were extracted from cardiomyocytes using TRIzol reagent (Invitrogen; USA). According to the manufacturer’s protocol, a total of 0.5 mg RNA was used to generate cDNA using the High-Capacity cDNA Reverse Transcription Kit (Applied Biosystems; USA). qRT-PCR was conducted under the following cycling conditions: 95°C/15 s, 60°C/30 s, and 72°C/30 s for 40 cycles, after an initial denaturation step at 95°C for 10 min; we used the SYBR Green PCR Master Mix (Applied Biosystems; USA) and amplification was performed on a 7500 Fast Real-Time PCR System (Applied Biosystems; USA). Transcript quantities were compared by applying the relative quantification method, as *GADPH* was used for template normalization. The relative value compared to the control sample was obtained using the 2^−ΔΔCT^ method. Table S1 shows the sequence details.

### Construction of a target crosstalk network (TCN)

The data of chemical-protein and protein-protein interactions is required to construct a single drug’s target association network and TCN for random pairwise drug combinations. In this study, the STITCH 3.1 database was chosen for retrieving verified drug-protein interactions for the drugs; as a confidence score, we used the threshold of 0.700 [25]. After importing the drug-protein interactions of a single drug into Cytoscape one by one, the Cytoscape plug-in Bisogenet was applied to retrieve experimentally validated protein-protein interactions from multiple datasets including BIOGRID, INTACT, MINT, DIP, BIND, and HPRD [27]. The isolated nodes representing proteins would be deleted in the target association network of a single drug. For construction of a TCN for a combination of 2 drugs, the target association networks of them must be established in advance, in which all of their network targets should be integrated. After that, only those nodes that show that a protein has a functional association with any network target belonging to another drug would be retained.

### Functional module analysis

The MCODE algorithm in the Cytoscape plug-in ClusterMaker [29] was chosen for searching for functional modules in a target association network of a single drug or in a TCN of a drug combination using the default settings of parameters. After uploading official gene symbols of all of the nodes in a target association network or TCN, the functional annotation tool DAVID [30] was then used to test whether these modules are significantly implicated in the gene ontology (GO) biological process ‘regulation of apoptosis’ (Accession ID: GO:0042981). Default tool options were applied. The apoptosis-regulatory module was established only if the false discovery rate (FDR) was less than 0.05. Finally, the proteins encoded by genes participating in ‘regulation of apoptosis’ were highlighted as yellow nodes in the networks of the apoptosis regulatory modules.

### Statistical analysis

All data are expressed as mean ± SEM. Analysis was performed using one-way ANOVA followed by Bonferroni’s test. Differences were considered statistically significant only if *p*<0.05.

## Supporting Information

Figure S1 **Effects of drugs on gene expression of** ***BAX*** **,** ***BAC2L1*** **,** ***BID*** **,** ***CASP8*** **, and** ***TP53*** **(A–E).** A concentration of 0.5 µM was used with each drug; the dose ratio was 1:1:1. ^#^ *p*<0.05, ^##^ *p*<0.01, ^###^ *p*<0.001 compared to the control group; n = 4; *p<0.05, **p<0.01, ***p<0.001 compared to the H_2_O_2_ group; n = 4; Cur: curcumin; Cel: celecoxib; Sil: silibinin.
(TIF)

Table S1 (DOC)
