# Supplementary figures and images for: Visualization of Network Target Crosstalk Optimizes Drug Synergism in Myocardial Ischemia
Source: PLoS One. 2014 Feb 5;9(2):e88137. doi: 10.1371/journal.pone.0088137 (PMC3914923; doi:10.1371/journal.pone.0088137)

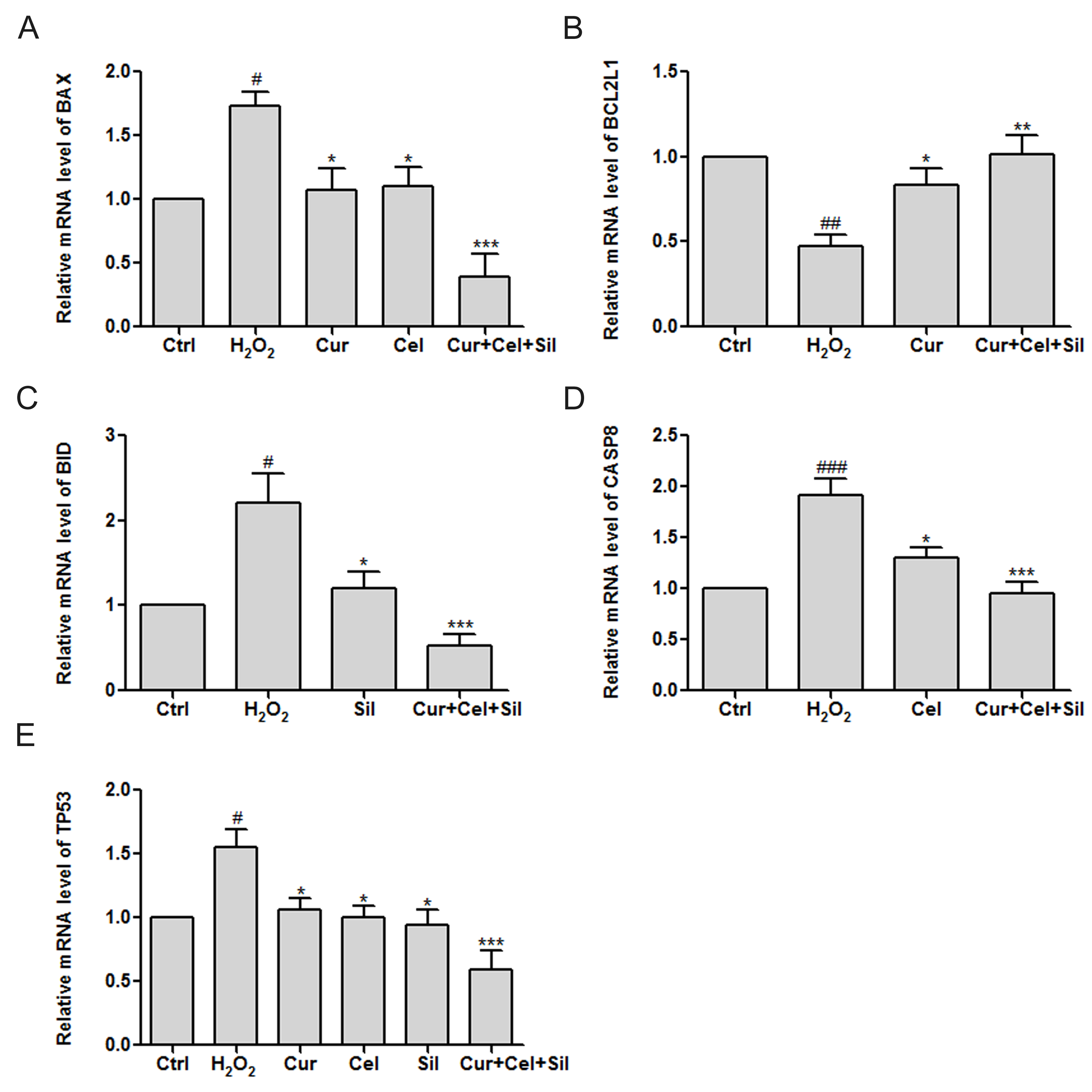

Supplement: Figure S1 — Effects of drugs on gene expression of BAX , BAC2L1 , BID , CASP8 , and TP53 (A–E). A concentration of 0.5 µM was used with each drug; the dose ratio was 1:1:1. # p<0.05, ## p<0.01, ### p<0.001 compared to the control group; n = 4; *p<0.05, **p<0.01, ***p<0.001 compared to the H2O2 group; n = 4; Cur: curcumin; Cel: celecoxib; Sil: silibinin. (TIF) [file pone.0088137.s001.tif]
