# Supplementary material for: Visualization of Network Target Crosstalk Optimizes Drug Synergism in Myocardial Ischemia
Source: PLoS One. 2014 Feb 5;9(2):e88137. doi: 10.1371/journal.pone.0088137 (PMC3914923; doi:10.1371/journal.pone.0088137)
Supplement: Table S1 — (DOC) [file pone.0088137.s002.doc]

**Table S1. Detailed sequences of the essential primers used in this study.**

| Gene | Sequence |
| --- | --- |
| BAX | Forward: 5′-GGTTATCTCTTGGGCTCACAAG-3′ |
|  | Reverse: 5′-TGATGGACGGGTCCGGGAGCA-3 |
| BCL2L1 | Forward: 5′- CAGAGCTTTGAACAGGTAG -3′ |
|  | Reverse: 5′- GCTCTCGGGTGCTGTATTG -3′ |
| BID | Forward: 5′-AAAACCACATGGCACAGAGA-3′ |
|  | Reverse: 5′-AGAGGGAACCACTTTGCTGA-3′ |
| CASP8 | Forward: 5′-GCTGTATCCTATCCCACG-3′ |
|  | Reverse: 5′-TCATCAGGCACTCCTTT-3′ |
| GAPDH | Forward: 5'-AAGAAGGTGGTGAAGCAGGC -3' |
|  | Reverse: 5'-TCCACCACCCAGTTGCTGTA -3' |
| TP53 | Forward: 5′-TTGCCGTCCCAAGCAATGGATGA-3′ |
|  | Reverse: 5′-TCTGGGAAGGGACAGAAGATGA-3′ |
